# Correction: Co-generation of NaREE(MoO_4_)_2_ and REEPO_4_ in multiple habits by solid-flux crystal growth

**DOI:** 10.1371/journal.pone.0342499

**Published:** 2026-02-10

**Authors:** Joseph Brian Balta, Megan E. Holycross, Buz Barstow, Esteban Gazel

The author Esteban Gazel should have been listed as a corresponding author. Esteban Gazel’s email address is: egazel@cornell.edu.
