# Supplementary material for: Co-generation of NaREE(MoO4)2 and REEPO4 in multiple habits by solid-flux crystal growth
Source: PLoS One. 2025 Nov 7;20(11):e0335161. doi: 10.1371/journal.pone.0335161 (PMC12594349; doi:10.1371/journal.pone.0335161)
Supplement: Attachment — Submitted filename: Revisioncommentsandreplies_MH2.docx [file pone.0335161.s003.docx]

When submitting your revision, we need you to address these additional requirements.

1. Please ensure that your manuscript meets PLOS ONE's style requirements, including those for file naming. The PLOS ONE style templates can be found at

<https://journals.plos.org/plosone/s/file?id=wjVg/PLOSOne_formatting_sample_main_body.pdf> and

[https://journals.plos.org/plosone/s/file?id=ba62/PLOSOne_formatting_sample_title_authors_affiliations.pdf](https://nam10.safelinks.protection.outlook.com/?url=https%3A%2F%2Ftrack.editorialmanager.com%2FCL0%2Fhttps%3A%252F%252Fjournals.plos.org%252Fplosone%252Fs%252Ffile%253Fid%3Dba62%252FPLOSOne_formatting_sample_title_authors_affiliations.pdf%2F1%2F010f01983d78f6c7-3933c093-613d-4680-b5a0-b5bb4e21ac8b-000000%2FXOBltOEZlryh5iS9w6SKhXw8524HFfhmIKFidkkIJZs%3D221&data=05%7C02%7Cjbalta%40lpi.usra.edu%7Cb979df1f9e444ed81c5508ddcad73dad%7C9f2aa711250341f08bb401fad7341a7b%7C0%7C0%7C638889748378120689%7CUnknown%7CTWFpbGZsb3d8eyJFbXB0eU1hcGkiOnRydWUsIlYiOiIwLjAuMDAwMCIsIlAiOiJXaW4zMiIsIkFOIjoiTWFpbCIsIldUIjoyfQ%3D%3D%7C0%7C%7C%7C&sdata=Mo5Z0UvbV7Ed5Z1v8KBroe45e%2BDX5ooRTo5R%2F6reXyQ%3D&reserved=0).

Thank you for providing this guide as I previously did not find this file and thus did not follow formatting as I didn’t know it. I have followed this formatting as well as I can, but I’m not 100% confident that the tiff file conversion that I used gave high enough quality images. I hope the journal will take a look at this, I have much larger files available but am trying to meet the requested slide size.

2. We note that the grant information you provided in the ‘Funding Information’ and ‘Financial Disclosure’ sections do not match.

When you resubmit, please ensure that you provide the correct grant numbers for the awards you received for your study in the ‘Funding Information’ section.

Thank you for this reminder. I will check the “Funding Information” section when resubmitting. It is possible that I did the best I could with a form online that was difficult to work with.

3. Thank you for stating the following financial disclosure:

 [This project was supported by ARPA-E award DE-AR0001341 to B.B, E.G., and M.E.H.].

Please state what role the funders took in the study.  If the funders had no role, please state: ""The funders had no role in study design, data collection and analysis, decision to publish, or preparation of the manuscript.""

If this statement is not correct you must amend it as needed.

Please include this amended Role of Funder statement in your cover letter; we will change the online submission form on your behalf.

Thank you for this reminder. This statement has been added to the cover letter. The statement was already present in the financial disclosure statement.

4. Please expand the acronym “ARPA-E” (as indicated in your financial disclosure) so that it states the name of your funders in full.

This is now complete.

This information should be included in your cover letter; we will change the online submission form on your behalf.

This is now complete.

5.  Please include captions for your Supporting Information files at the end of your manuscript, and update any in-text citations to match accordingly. Please see our Supporting Information guidelines for more information: <http://journals.plos.org/plosone/s/supporting-information>.

This is now complete.

6. If the reviewer comments include a recommendation to cite specific previously published works, please review and evaluate these publications to determine whether they are relevant and should be cited. There is no requirement to cite these works unless the editor has indicated otherwise. 

Thanks for this explanation. All of the reviewers’ recommended citations have been included.

[Note: HTML markup is below. Please do not edit.]

Reviewers' comments:

Reviewer's Responses to Questions

**Comments to the Author**

1. Is the manuscript technically sound, and do the data support the conclusions?

The manuscript must describe a technically sound piece of scientific research with data that supports the conclusions. Experiments must have been conducted rigorously, with appropriate controls, replication, and sample sizes. The conclusions must be drawn appropriately based on the data presented.

Reviewer #1: Yes

Reviewer #2: Yes

2. Has the statistical analysis been performed appropriately and rigorously?

Reviewer #1: N/A

Reviewer #2: N/A

3. Have the authors made all data underlying the findings in their manuscript fully available?

The [PLOS Data policy](https://nam10.safelinks.protection.outlook.com/?url=http%3A%2F%2Ftrack.editorialmanager.com%2FCL0%2Fhttp%3A%252F%252Fwww.plosone.org%252Fstatic%252Fpolicies.action%2523sharing%2F1%2F010f01983d78f6c7-3933c093-613d-4680-b5a0-b5bb4e21ac8b-000000%2Fb4YbTNfsbKByUiIRSRymJB7IQSxukq94M-v-48IeyMU%3D221&data=05%7C02%7Cjbalta%40lpi.usra.edu%7Cb979df1f9e444ed81c5508ddcad73dad%7C9f2aa711250341f08bb401fad7341a7b%7C0%7C0%7C638889748378131250%7CUnknown%7CTWFpbGZsb3d8eyJFbXB0eU1hcGkiOnRydWUsIlYiOiIwLjAuMDAwMCIsIlAiOiJXaW4zMiIsIkFOIjoiTWFpbCIsIldUIjoyfQ%3D%3D%7C0%7C%7C%7C&sdata=EE1j91BtJHcAJMP21WdRoYWs2ha4Rpbrkq1onFwxMZk%3D&reserved=0) requires authors to make all data underlying the findings described in their manuscript fully available without restriction, with rare exception (please refer to the Data Availability Statement in the manuscript PDF file). The data should be provided as part of the manuscript or its supporting information, or deposited to a public repository. For example, in addition to summary statistics, the data points behind means, medians and variance measures should be available. If there are restrictions on publicly sharing data—e.g. participant privacy or use of data from a third party—those must be specified.

Reviewer #1: Yes

Reviewer #2: Yes

4. Is the manuscript presented in an intelligible fashion and written in standard English?

PLOS ONE does not copyedit accepted manuscripts, so the language in submitted articles must be clear, correct, and unambiguous. Any typographical or grammatical errors should be corrected at revision, so please note any specific errors here.

Reviewer #1: Yes

Reviewer #2: Yes

5. Review Comments to the Author

Please use the space provided to explain your answers to the questions above. You may also include additional comments for the author, including concerns about dual publication, research ethics, or publication ethics. (Please upload your review as an attachment if it exceeds 20,000 characters)

Reviewer #1: Comments on the manuscript "Co-generation of NaREE(MoO4)2 and REEPO4 in multiple habits by solid-flux crystal growth".


The main goal of the paper is to refine the lab conditions for REE-inorganic phases synthesis. The focus is on the synthesis of REE phosphate (monazite) and of sodium-molybdenum-REE oxide (NaREE(MoO4)2) that has several industrial applications.
To synthesize the REE-oxide, the strategy was to separate REE from a REE-bearing phase (artificial Ce, Nd, Gd, Yb, La phosphates or natural monazite) using a mixture of an oxide (MoO3) and a carbonate (Na2CO3, LiCO3) as a flux, and subsequently varying i) the proportions of oxide:carbonate and phosphate:flux, ii) the temperature of loading, permanence and removal from heating in furnace, and iii) cooling in fast or slow conditions.
To achieve their goal, the authors based their research on previously published lab procedures and carried out four experiment types with successive degrees of improvement in procedures and results. Experiments 1 and 2 resulted in learning about the materials and mixtures used, the best way to heat and cool the samples, and the safe use of experimental apparatus (e.g., crucible, furnace). Experiment 3 had success in crystallizing up to 5 mm monazite. Experiment 4 was distinguished by its use of natural monazite as starting material and heat treatment at high pressures in a piston-cylinder apparatus and was successful in synthesizing large monazite surrounded by lathes of NaREE(MoO4)2.
The authors also provided updated recommendations for safer lab procedures and for synthesizing larger REEPO4 crystals of interest to the industrial sector.
The text is well-written and perfectly understandable. A few typing mistakes and punctuation errors were found (e.g., a full stop in line 139, a semicolon in line 225, and parentheses in line 589).

Thanks for this recommendation. I have fixed the parenthesis at line 589 and a spelling mistake at line 140, revaluated the punctuation at lines 139 and 225.

The four experiment types and new results are described in detail in the text. Samples were characterized by optical microscopy, Raman spectroscopy, XRD, electron microprobe, and laser ablation ICP-MS. SEM images of good quality illustrate the crystallized phases. The data provided by the study is of good quality and supports interpretations and conclusions.
The article is essential for improving the knowledge about the methods that can separate REE from minerals such as monazite, an abundant mineral containing REE. Thus, the manuscript fits the scope of the PLOS ONE journal. However, I would recommend minor modifications and clarifications before publication, as listed below.

Minor recommendations to the manuscript:
1) In line 146, “project” could be changed to “study, investigation”.
Thanks for this recommendation. I used “investigation”.
2) In line 152, “presentation” could be changed to “paper”.
This change is now complete.
3) The minerals mentioned in the captions of figures 4 and 5 could be labeled in the images.
Thanks for this recommendation. In figure 4, there are only two phases present as described in the caption. I added small lines labeling the needle-like morphologies.

Figure 5 has been labeled, thank you.

Reviewer #2: I read your manuscript and think it brings some news to the subject and may be of interest to a significant audience, after some moderate/major optimization. I think your experimental and analytical data are mostly ok and your main conclusions seems to sound mostly well. Congrats! Please find in the below some suggestions/corrections and comments. Hope they help to improve the presented version. I am not native so apologize for any English Language fault.

Silvio RF Vlach

General: not a big deal, however normally the used abbreviations are explained in the first time they appear on the text; e.g. Electron Probe Micro Analyzer (EPMA).

Thanks for this suggestion. EPMA is defined as Electron Microprobe at line 360.

Please do not mix methods with results and/or discussion (e.g., Materials and Methods, Experiment type three: Successful phosphate synthesis)
Thanks for this recommendation. One line was removed from the type 3 section describing crystal morphology.

Methods: the standards used for EPMA quantification need to be better referred; indicate also the method used for spectral treatment and matrix effect corrections as well as the expected detection limits/analytical errors. These latter need to be also informed in the section on LA-ICP-MS analysis.
Thanks for this recommendation. I agree, it is best practice to report the analytical data reduction treatment in full. However, the microprobe outputs I have in hand do not reported the correction method used, so I can’t include it. I suspect it is the ZAF correction though.

I have added the specified standards, and a brief discussion of detection limits and standard deviations. I have also added all of our EPMA analyses including the full analysis details as output from the probe to the supplementary information.
When you mention flux : REEPO4 ratios please add in the table and at the first mention in the text if this ratio refers to weigh or molar ratios. How is the precision of the used analytical balance?
Thanks for this suggestion. This ratio is given in Table 1. The requested detail in the text has been added, as has a note for how to find this ratio in the table.

The balance I used for this project reports masses to 0.00001. The stated precision of the scale is 1 mg at masses <50 g. The limitation on the measured weights has more to do with human error-- I cannot precisely move material in increments this small, and sometimes material adheres to weigh paper and weigh boats. In general, I estimate my errors are ~0.001 g or less based on the measurements of material left behind on weigh paper and the target weights.

Results: the audience would like to see some representative compositions of the products (REEPO4 and NaREE(MoO4)2 crystals from the main experiments within the main text. Also how your LREE, P, Mo and Na analytical results from EDS compares with those from LA-ICP-MS? In general WDS (and so I think probably EDS) results for the major elements are better than those obtained with LA-ICP-MS. Along with such compositions, please add the relevant cationic proportions in relation to the appropriate Oxygen numbers to demonstrate the stoichiometry of your products. This will reinforce your XRD results and allow the audience to validate your analytical results on the main crystalline products.

Thanks for this recommendation. Full analyses of crystals from numerous experiments were measured on the LA-ICP-MS and have been included in the supplementary information.

No analysis that has been reported in this work contains oxygen as an individually measured component. It is not measured in the LA-ICP-MS analyses (oxygen is not measurable with our instrument) and was calculated by stoichiometry on the EDS and the electron microprobe. Thus we do not have the ability to independently verify or reinforce our XRD results with a direct measurement.

Select EDS analyses on pure Ce crystals are included in supplementary table 1. The variance on these analyses is so high that we do not endorse these analyses for anything other than verifying the phase as a phosphate in a quick analysis. We would recommend the same for the LA-ICP-MS analyses, the variance on these analyses is just too high to use them for specific comparisons of one atom to another other other than to say what was present. For example, in the reported materials, EDS analyses have Ce contents vary from 8 to 11% when reported as a metal, which is likely much higher than the actual difference from one crystal to another but is almost certainly reflecting the cracked and difficult to work with nature of the material. Similarly, we have not rigorously compared measurements from one tool to another, but as can be seen in the supplementary tables, the variance on each analysis type was very high, any sort of comparison attempt like this would not be likely to produce a useful product. I have added the full electron microprobe table output from analyses on experiment type 4 to the supplementary table.

You mention the use of the Raman spectroscopy but I did not see any results and the relevance of this method for them.
The Raman spectrometer was used to characterize starting materials for experiment type 4. The text reads: “This material was verified to be monazite using a WiTec Alpha 300r Raman spectrometer (Section 2.5.1).”

We went all the way back to the original files and tracked down these spectra, one of which is monazite and one of K-Feldspar.

I believe the main peaks in this shot indexed as monazite.

OK, the used flux avoids Pb contamination, but how about Mo contamination? If there is some, how is the advantage of the method in the case of synthesized REEPO4 standards?

Thanks for pointing this out. For the purposes of the experiments, avoiding working with lead, due to health hazards, was a major benefit. Our LA-ICP-MS analyses (supplementary table) show a range of low Mo contents in this work, suggesting Mo contamination in the experiments was negligible.

Line 48: please, the so-called rare earth elements include, besides the lanthanides, Sc and Y (see IUPAC).

Thanks for this recommendation, the change is now complete.

Line 53: think REE-bearing is better

Thanks for this recommendation, the change is now complete.

Line 54-55: please, basnäsite is a LREE fluorocarbonate. I think basnäsite does not contain significant amounts of Th, U, and, so, Pb. PLease check!

Thanks for this suggestion. My survey of the literature suggests the amount of Th, U and Pb in banasite depends on the deposit. For example, the Mountain Pass mine in California, USA, where basnasite is mined, has had significant issues with thorium spills during its operational time. It is also notable that this mine also was originally prospected for as a uranium deposit, so while the U concentrations may not be excessive in bastnäsite, I believe this statement is accurate.

Line 58: (a rock or mineral with economic, etc.)

Thanks for this recommendation, the change is now complete.

Lines 95-96. I think you should also take a look in Donovan et al. (2002), J. Res. Natl. Inst. Stand. Technol., 107, and references therein for REE phosphate synthesis and Pb contamination trough Pb-bearing fluxes.

Thanks for this recommendation, the change is now complete.

Line 111: are they all crystal habits or include crystal aggregate habits?
Thanks for this recommendation. At least one is an aggregate so we have now changed the wording.

Line 139. …carbonate flux. (period) If this…

Thanks for this recommendation, the change is now complete.

Line 166: please extend the table caption. Add P/F ratio = Phosphate/Flux (molar or weight) ratio in it. Specify better the experiments carried out with a furnace and a piston-cylinder. Add the simulation pressure in the latter case.

Thanks for this suggestion. The caption has been extended, and it was defined in the caption that the type 1-3 experiments were 1 atmosphere and type 4 was in a piston cylinder.

Line 343. Raman spectroscopy and X Ray Diffractometry sounds better. Analogous for the other presented methods.

Thanks for this suggestion. We used X-Ray diffraction, but have now used the full words rather than the abbreviation.

Line 362. I think the readers would like to see some EDS compositional maps.

Thanks for this recommendation. The EDS maps we collected were not high quality and were done solely to assist with locating phases before microprobe analyses. In our opinion, they are not of publication quality, so we choose to leave them out. An example EDS compositional map is provided below.

Line 364: please specify that the quantification was done with EDS (rather than WDS) analysis.

Thanks for this recommendation, the change is now complete.

Line 635. Is Na3PO4 a reliable compound? Please check!
Thanks for this suggestion. Trisodium phosphate is a common food additive, so it is stable at room conditions. We suggest Na3PO4 was formed because the phosphate and most of the sodium were not found after the experiments – they were removed in an open system process to allow the reaction to proceed. The exact species that contained these phases is unknown, but this is a good potential representative.

A note has been added to clarify that this component is standing in for material that was never found when the reaction ran to completion.

Line 642. Of note, the transition to a xenotime structure is significantly related to the cationic radii and should be expected in all REEPO4 compounds where REE has a relatively small radius, approaching the Y radius (as the most heavy lanthanides).

Thanks for this note. We do not observe a transformation from the monazite structure to the xenotime one, it’s a transition from the rhabdophane or hexagonal structures to the monazite/xenotime structures. We do not probe the transition from monazite to xenotime within a single experiment.

Line 654. I missed something here: “intermediate hexagonal structure”?

. [37] noted that the hydrous, hexagonal REE phosphate rhabdophane forms when precipitation from solution is done below 90°C, as in all our synthesis reactions. They then showed that rhabdophane transitions to an anhydrous hexagonal phase between 100 and 400°C and then to true monazite at temperatures as low as 400°C for LaPO_4_, but as high as 900°C for DyPO_4_.

This clause was above with a citation, currently line 343 (but that’s after some edits). I have added an additional note to highlight this for the xenotime transition and added the word “Intermediate” as well to try to make sure it wasn’t missed.

Line 683. I think the industrial potential and eventual drawbacks should be more elaborated in this paragraph.

Thanks for this recommendation, the change is now complete. The additional lines read:

While this conversion does not deal with contaminants such as U or Th, and additional steps may be required to remove the transition metal from the final oxide, previous literature suggests that the inability to break down monazite has been a major impediment to its use as an REE source. It is possible that this method, roasting in the presence of a transition metal such as molybdenum, could be a method of processing either recycled REE or minerals that are typically difficult to access, as shown here with monazite.

Line 695: revise phrasing.

Thanks for this recommendation, the change is now complete.

Lines 701-705. In some place along the text put some statements/comparisons on the relations between the obtained/known crystal habits and experimental conditions. Certainly there is a significant one.

Thanks for this recommendation, the change is now complete. The additional text in the conclusion now reads:

“We also showed this material forms skeletal and a needle-like habits; while numerous morphologies have been demonstrated in the literature for these materials, the other morphologies were commonly produced in small quantities and from synthetic materials. In contrast, our technique produced substantial quantities of NaREE(MoO_4_)_2_ and could be applied for conversion of natural materials into this oxide. NaREE(MoO_4_)_2_ has numerous industrial applications, and our experiments produced substantial amounts of material with large reactive surface areas; while many of the previous methods produced reactive surface areas, this technique offers the advantage of working with available natural starting materials and produces material without sacrificing the reactive surfaces that could be used for catalysis. Consequently, the methods detailed here could be used to produce NaREE(MoO_4_)_2_ quickly and in sufficient quantity for industrial use.

Figures

Figure 1 caption: Ok, Nd-monazite crystallizes in the monoclinic system, however I think you cannot conclude this just on the basis of the habit of the shown crystal; a similar crystal should have also a triclinic symmetry. The last phrase is unnecessary.

Thanks for this note. I added a detail that the symmetry was not the only way that this phase was confirmed, once we found it, we also verified via EDS that this was a phosphate.

Figure 2. I think XRD diffractogram/spectrum is better than XRD analyses. Sorry, I don’t remember: is it ok to indicate (1,1,10), with commas, as a Miller index? Please check.

Thanks for this suggestion. While generating this figure, we matched the format that was output by the XRD software. We made it look better but kept the text formatting.

Figure 3 caption: The samples L to R cannot be properly seen in (a); add letters below the respective containers. There is no tweezers in (b), just a conventional scale.

Thanks for this recommendation, the change is now complete.

Figure 4. please specify: SE (secondary electron) images. I think you could say more based on them, concerning the observed crystalline habits. In (d) it appears you have a skeletal crystal (?). I am not sure if the NOTE is necessary.

Thanks for this suggestion. We have now specified that these were inlens detector images and removed the note.

Figure 5. Please identify (annotate) each phase over the BSE images.

Thanks for this recommendation, the change is now complete.


Supplementary Materials

Please, optimize the organization and formats of your numeric tables to facilitate readers/potential users.

Thanks for this suggestion. We have reevaluated the table and found the format is optimal for our purposes. We have also added a table S5 containing the raw microprobe analyses.

References

I did not check all in detail. However check the above suggested references.

References have been checked and formatted.
